# Ileocecal Adenocarcinoma and Ureteral Transitional Cell Carcinoma with Multiple Sebaceous Tumors and Keratoacanthomas in a Case of Muir-Torre Syndrome

**DOI:** 10.1155/2010/173160

**Published:** 2010-08-15

**Authors:** Michael C. Lynch, Bryan E. Anderson

**Affiliations:** Department of Dermatology, Penn State College of Medicine, 500 University Drive, Hershey, PA 17033-0850, USA

## Abstract

Cutaneous neoplasms including sebaceous tumors, keratoacanthomas, and basal cell carcinomas with sebaceous differentiation can be markers of internal malignancy associated with the Muir-Torre Syndrome (MTS). We report a 56-year-old man with a diagnosis of hereditary nonpolyposis colorectal cancer (HNPCC) and ureteral transitional cell carcinoma who subsequently developed two sebaceous gland neoplasms and several keratoacanthomas, leading to the diagnosis of MTS. Our case highlights the clinical advantages of immunohistochemistry (IHC) in identifying mutations in the mismatch repair (MMR) genes responsible for both HNPCC and MTS. The importance of continued clinical suspicion in the dermatological assessment of patients with sebaceous neoplasms is emphasized.

## 1. Introduction

Usually diagnosed clinically by the synchronous or metachronous manifestation of a sebaceous neoplasm and visceral malignancy, most commonly colon cancer, MTS was first described by Muir et al. in 1967 and subsequently by Torre in 1968 [1, 2]. Later, Lynch et al. described several patients with sebaceous neoplasms, colorectal cancer, and a familial cancer predisposition and first considered MTS as an uncommon phenotypic variant of HNPCC [3]. 

Although MTS is a rare, autosomal genodermatosis, the serious but curable nature of its associated cancers makes early identification paramount. In such, cutaneous neoplasms can serve as visible markers of internal malignancy. The most important of these being sebaceous adenoma, a relatively rare but benign tumor that presents as yellow papules or nodules and acts as the most specific marker of MTS [4, 5].

## 2. Case Report

Our patient first presented with a change in bowel caliber and fatigue. During his interview, a significant family history of cancer was identified with five of his ten siblings diagnosed with a variety of cancers. Specifically, a sister diagnosed with colon cancer, a brother diagnosed with kidney cancer, another brother diagnosed with two colon cancers, a third brother diagnosed with two colon cancers and skin cancer, and a fourth brother diagnosed with two colon cancers, ureter cancer, and skin cancer. Additionally, his father was diagnosed with colon cancer in his late forties and his paternal uncle was diagnosed with colon cancer. 

With a presentation and family history consistent with HNPCC, he was initially evaluated with a pelvic-computed tomography (CT) scan. After visualization of a 3.5 × 5.5 cm mass near the ileocecal valve, an endoscopy-guided biopsy of the mass was performed. Pathology characterized the mass as an adenocarcinoma with moderate differentiation. A subtotal colectomy followed which confirmed the biopsy results. No serosal invasion or lymph node involvement was noted. Following the colectomy, he elected to pursue research-based testing of the MSH2 and MLH1 genes, the most common MMR proteins implicated in HNPCC [5]. The results of this study however were several years away, and in the meantime an increased cancer surveillance program for the patient and his family members was recommended.

A year later, he noticed hematuria and a lower abdominal mass. A pelvic CT was ordered which showed right hydroureteronephrosis, but no external mass. This was followed up with an intravenous pyelogram (IVP) and urinary cytology. The IVP confirmed the hydroureteronephrosis and cytologic examination showed numerous small, atypical cells with a high nuclear-cytoplasmic ratio highly suspicious for malignancy. A low-grade urothelial carcinoma was found in the right ureter and a right nephroureterectomy was performed. 

With no results forthcoming in the research-based testing of MMR genes and a growing concern for other family members, he decided to pursue clinical genetic testing. DNA sequencing for both MSH2 and MLH1 was undertaken. However, no explainable mutation was discovered and uncertainty remained. 

The patient presented to our dermatology clinic with concerns of a flesh-colored papule on his left cheek. Surgical excision and skin biopsy were performed and identified a sebaceous adenoma. With the presentation of this sebaceous neoplasm and his history of visceral malignancy, a new diagnosis of MTS was made. The following year, a similar lesion was removed from his chin and biopsy identified a keratoacanthoma. 

Still without the identity of the causative mutation, he decided to pursue additional clinical genetic testing utilizing new Southern blotting technology in the screening of MSH2 and MLH1. This study finally identified a deletion of exons 1 through 7 in the MSH2 gene. With an explanation for his personal cancers and family history, genetic testing was offered to his extended family. 

**Figure 1:**
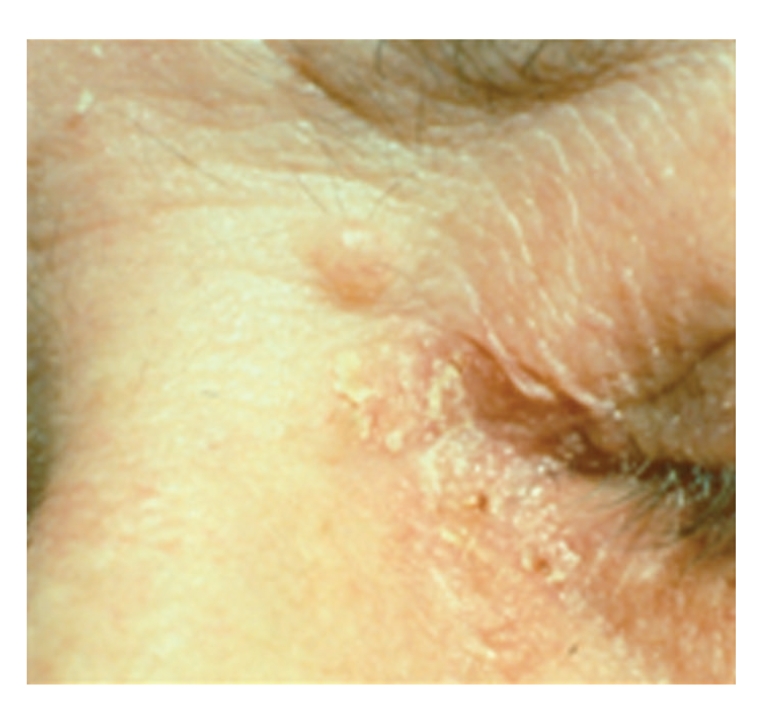
Sebaceous carcinoma of eyelid found during routine screening of a patient with Muir-Torre Syndrome.

Since the mutation has been identified, he has undergone regular colonoscopies, urinary cytologic analysis, dermatologic assessment, and monitoring of carcinoembryonic antigen (CEA). Colonoscopy and urinary cytology have been negative and CEA levels have been stable. Subsequent biopsies of a variety of skin papules have confirmed a sebaceous carcinoma and a second keratoacanthoma (Figure 1).

## 3. Discussion

Although MTS generally presents as a sebaceous neoplasm plus colorectal adenocarcinoma, a secondary diagnostic criterion includes multiple keratoacanthomas associated with visceral neoplasms and a family history of MTS [5]. The internal cancers implicated include carcinomas of the genitourinary tract, breast, upper gastrointestinal tract, larynx, parotid gland, and hematological malignancies. Of these, tumors of the genitourinary tract (especially ureter and endometrium) are the most common visceral neoplasms seen in MTS after colorectal cancer [4]. 

Cancer surveillance and early identification of these tumors are important for the implementation of proper treatment regimens. Because sebaceous tumors can serve as visible markers of internal malignancy in MTS, a detailed dermatologic exam is important in these patients. Sebaceous hyperplasia and solitary keratoacanthoma, relatively common findings in the general population, are not considered markers of MTS, but all other sebaceous neoplasms, including sebaceous adenoma, sebaceoma, and carcinoma, are considered highly specific markers of MTS [5]. 

Sebaceous adenoma, the most common lesion in MTS, presents as tan or yellow neoplasms that contain a spectrum of cells ranging from germinative or basaloid to fully mature sebocytes. Pleomorphism is exceptional in cases of sebaceous adenoma [6]. Sebaceomas show a more prominent basaloid component and are probably more easily confused with basal cell carcinoma or sebaceous carcinoma. Their basaloid content is greater than 50% of cellularity by definition [6]. Sebaceous carcinoma, a malignant tumor, has two distinct variants: an aggressive form that occurs most frequently on the eyelids and an extraocular type, whose aggressiveness is still debated, that occurs most commonly on the head and neck [7]. In MTS, the extraocular form is the usual presentation. 

Whenever multiple sebaceous tumors are identified involving any site or any sebaceous tumor is discovered outside of the head and neck region, especially in a young person (<50 years), a thorough work-up for MTS is necessary. The initial test should be IHC, using antibodies to bind specific cellular antigens, to determine the presence or absence of MMR proteins [5]. 

MMR genes encode proteins that recognize and remove improper genomic insertions and deletions, and nucleotide mismatches that arise during DNA replication and recombination. Collectively these genes maintain the integrity of the genome and prevent mutations from accumulating. In MTS, individuals are born with one defective copy of an MMR gene and acquire a second somatic mutation in the other allele during their lifetime [5]. Interestingly, it has been shown that MMR deficiency is more likely to be found in benign sebaceous tumors as compared to sebaceous carcinoma, and more common in lesions that occur outside the head and neck [7]. 

The first mutations identified in MTS involved the MMR genes MSH2 and MLH1, the same genes known to cause HNPCC. This insight has led MTS to be considered a phenotypic variant of HNPCC [3, 8]. Subsequent studies have shown that mutations in MSH2 are the most common, comprising about 90% of MTS cases, and that about 10% of patients have mutations in MLH1 [8]. Recently however, individuals have also been identified with MTS phenotype but with mutations in MSH6 [9, 10]. This discovery has added complexity to the MTS genotype, but offers an additional gene for mutation analysis. 

IHC, assessing MSH2, MLH1, and MSH6 activity, and microsatellite instability (MSI) analysis are currently the two main screening tests for MTS. Using a combination of these tests, most cases of MTS should be identified after a thorough clinical examination and family history. The recent advances in IHC and MSI analysis have made the diagnosis of MTS much simpler.

Although screening MMR genes for mutations can detect MTS genotypes, the protracted nature of these tests and the fact that not all causative mutations are known make these mutation screenings a poor first diagnostic choice. The shortcomings in mutation screenings are highlighted by the difficulty our patient had in identifying a mutation and the many years before this technique obtained clear results. 

Because early diagnosis of MTS is essential for both the patient and their extended family, we believe that mutational screenings are not the best initial diagnostic approach. We agree with recent proposals to use IHC as the initial screening test assaying MSH2, MLH1, and if both of these are negative MSH6 [5]. MSI analysis and review of family history should guide cancer surveillance options for the patient and extended family after IHC. Mutational analysis should be considered only as a confirmation step and to screen family members for necessity of continued cancer surveillance. 

It is the responsibility of dermatologists to carry a strong suspicion when evaluating sebaceous tumors, especially those with an unusual presentation. The proper tests and early detection of malignancies will hopefully allow patients with MTS to reach normal life expectancies.

## References

[1] Muir EG, Bell AJ, Barlow KA (1967). Multiple primary carcinomata of the colon, duodenum, and larynx associated with kerato-acanthomata of the face. *British Journal of Surgery*.

[2] Torre D (1968). Multiple sebaceous tumors. *Archives of Dermatology*.

[3] Lynch HT, Lynch PM, Pester J, Fusaro RM (1981). The cancer family syndrome: rare cutaneous phenotypic linkage of Torre’s syndrome. *Archives of Internal Medicine*.

[4] Ponti G, de Leon MP (2005). Muir-Torre syndrome. *Lancet Oncology*.

[5] Abbas O, Mahalingam M (2009). Cutaneous sebaceous neoplasms as markers of Muir-Torre syndrome: a diagnostic algorithm. *Journal of Cutaneous Pathology*.

[6] Shalin SC, Lyle S, Calonje E, Lazar AJF (2010). Sebaceous neoplasia and the Muir-Torre syndrome: important connections with clinical implications. *Histopathology*.

[7] Singh RS, Grayson W, Redston M (2008). Site and tumor type predicts DNA mismatch repair status in cutaneous sebaceous neoplasia. *American Journal of Surgical Pathology*.

[8] Wu CY (2009). Muir-Torre syndrome: extraocular sebaceous carcinoma with adenocarcinoma of colon in a 76-year-old man. *Clinical and Experimental Dermatology*.

[9] Arnold A, Payne S, Fisher S (2007). An individual with Muir-Torre syndrome found to have a pathogenic MSH6 gene mutation. *Familial Cancer*.

[10] Murphy HR, Armstrong R, Cairns D, Greenhalgh KL (2008). Muir-Torre syndrome: expanding the genotype and phenotype—a further family with a MSH6 mutation. *Familial Cancer*.

